# MS4A7 based metabolic gene signature as a prognostic predictor in lung adenocarcinoma

**DOI:** 10.3389/fmolb.2025.1591446

**Published:** 2025-04-28

**Authors:** Yan Jiang, Zhengyu Shu, Lei Cheng, Haowei Wang, Taiping He, Liwen Fu, Chao Zhao, Xuefei Li, Weicheng Zeng

**Affiliations:** ^1^ Department of Reproductive Medicine Nursing, Key Laboratory of Birth Defects and Related Disease of Women and Children, West China Second University Hospital, Sichuan University, Chengdu, China; ^2^ Jiangsu Province Hospital of Chinese Medicine Chongqing Hospital, Chongqing Yongchuan Hospital of Chinese Medicine, Chongqing, China; ^3^ Department of Lung Cancer and Immunology, Shanghai Pulmonary Hospital, School of Medicine, Tongji University, Shanghai, China; ^4^ Department of Orthopedic Oncology, Shanghai Bone Tumor Institute, Shanghai General Hospital, Shanghai Jiao Tong University School of Medicine, Shanghai, China

**Keywords:** MS4A7, prognosis prediction, lung adenocarcinoma, tumor microenvironment, metabolism-related genes

## Abstract

**Background:**

Lung adenocarcinoma (LUAD) represents the most common form of lung cancer, contributing to significant global mortality. Metabolic reprogramming in tumor cells has been increasingly recognized as a hallmark of tumorigenesis, contributing to an immunosuppressive microenvironment. Given the promising prediction value of metabolism-related genes in LUAD, this study aims to explore the role of MS4A7, a member of the MS4A gene family, in LUAD prognosis and immune microenvironment dynamics.

**Methods:**

A prognostic signature for LUAD was developed using the LASSO-Cox regression algorithm with RNA-seq data from 500 LUAD patients in The Cancer Genome Atlas database. Genes with differential expression linked to metabolic pathways were identified, and 20 genes were included to develop a risk signature. Further functional enrichment analysis was conducted to compare the biological pathways activated in high-risk versus low-risk groups. Single-cell RNA sequencing was employed to identify the expression profile and role of MS4A7 in different macrophage populations within the LUAD.

**Results:**

The constructed prognostic model displayed high predictive accuracy, outperforming single gene-based predictions. High-risk patients exhibited significantly poorer survival outcomes. Pathway enrichment analysis revealed dysregulated metabolic pathways in high-risk patients, including activation of glycolysis, mTORC1 signaling, and ROS production. Single-cell RNA sequencing revealed that MS4A7 expression was predominantly found in macrophage populations, with high expression localized in MS4A7+ macrophages. These macrophages exhibited distinct metabolic reprogramming and key immune functions, particularly in crosstalk with T cells and neutrophils.

**Conclusion:**

The MS4A7 gene plays a critical role in LUAD prognosis, particularly through its involvement in immune modulation within the TME. MS4A7^+^ macrophages, characterized by distinct metabolic reprogramming and immune interactions, are pivotal in shaping LUAD progression and immune response. The findings highlight the potential of MS4A7 as a novel prognostic biomarker and therapeutic target for LUAD. Further investigation into the metabolic and immune regulatory mechanisms of MS4A7^+^ macrophages could offer new insights into LUAD treatment strategies.

## Introduction

Lung cancer, a global health priority, led to 1.79 million deaths in 2020 (Sung et al., 2021). Among the various subtypes of lung cancer, lung adenocarcinoma (LUAD) has emerged as the most prevalent, representing over half of all lung cancer diagnoses (Chen et al., 2018; Chen et al., 2022; Chen et al., 2024; Wei et al., 2023). LUAD progression is recognized as a multistep process, transitioning from atypical adenomatous hyperplasia and adenocarcinoma *in situ* to microinvasive and invasive adenocarcinoma, as supported by histological, cytological, and molecular findings (Succony et al., 2021). Genetic mutations, such as alterations in the epidermal growth factor receptor, P53, Kirsten rat sarcoma, and mesenchymal-epithelial transition genes, play critical roles in the development and progression of LUAD (Shaurova et al., 2020; Wang et al., 2021; Wang et al., 2023; Li et al., 2022).

A hallmark of cancer is metabolic reprogramming, a phenomenon that has garnered significant attention in recent decades (Hanahan, 2022). Research into the metabolism of cancer cells and their surrounding tumor microenvironment (TME) has revealed the potential of targeting metabolic pathways to enhance anti-cancer immunity (Bader et al., 2020). Metabolism gene in tumor cells can lead to the accumulation of metabolites within the TME, or cause nutrient competition between tumor and stromal cells (Spella and Stathopoulos, 2021). This can result in an immunosuppressive TME, which contributes to poor prognosis in patients. Therefore, metabolism-related genes (MRGs) show promise as prognostic markers for LUAD.

The MS4A gene family, consists of at least 16 members, are crucial in cell differentiation, signaling, and the regulation of the cell cycle (Mattiola et al., 2021; Ni et al., 2023). Several MS4A family members, including MS4A1/3/4/6/7/12/15, have been linked to cancer onset and progression, although the exact mechanisms not being fully understood (Pan et al., 2020; Mudd et al., 2021; Luo et al., 2022; Zhao et al., 2022). Among them, MS4A2 has been closely linked to the prognosis of lung adenocarcinoma (Silva-Gomes et al., 2022) and brain metastases (Chen et al., 2021). On the other hand, MS4A8 appears to influence cellular development of LUAD (Kudoh et al., 2020; Zhou et al., 2024), while the role of MS4A7 in LUAD remain under-explored.

In this research, we developed a prognostic signature index for LUAD, grounded in MS4A7. We also examined the molecular and cellular signature differences between high and low-risk subgroups, along with their immune infiltration profiles. Additionally, we employed single-cell RNA sequencing (scRNA-seq) analysis to elucidate the cell types associated with these signature genes.

## Methods

### Data collection and preprocessing

RNA-seq data and clinical information for 500 LUAD patients were obtained from The Cancer Genome Atlas (TCGA) database. The dataset was preprocessed to remove missing or incomplete clinical data and to ensure that only appropriate samples were considered in the analysis. Patients with incomplete or missing follow-up data were excluded. The RNA-seq expression data were log-transformed and normalized to ensure comparability across samples. Additionally, data on survival time and other clinical variables such as age, gender, smoking history, and tumor stage were retrieved and integrated with the gene expression profiles.

Differentially expressed genes (DEG) between normal and tumor tissues were identified using the “edgeR” package in R. For this, raw counts of RNA-seq data were filtered to retain genes with an average expression level greater than one across all samples. A p-value <0.05 and fold-change ≥1 were used as criteria to define significantly differentially expressed genes. These DEG were then mapped to the Kyoto Encyclopedia of Genes and Genomes (KEGG) metabolic pathway database to identify genes involved in metabolic pathways that may play a role in LUAD progression.

### Development of a metabolism-related gene risk signature

To develop a prognostic signature, we performed the Least Absolute Shrinkage and Selection Operator (LASSO) Cox regression method. A total of 20 candidate genes linked to metabolic pathways were selected based on their differential expression and involvement in LUAD pathogenesis.

The prognostic performance of the model was evaluated by calculating the area under the curve (AUC) of the receiver operating characteristic (ROC) curve for the risk signature. Patients were classified into high and low-risk subgroups based on the median score. Kaplan-Meier survival analysis was used to assess the overall survival (OS) of patients in the groups. The Cox proportional hazards regression model was employed to assess the independent prognostic value of the risk score in combination with other clinical factors.

### Gene Set Enrichment Analysis

To identify biological pathways that were significantly enriched in LUAD patients, we performed Gene Set Enrichment Analysis (GSEA). Genes from the Hallmarker genesets were used to examine the activation of various metabolic and immune-related pathways. The top 10 most significantly enriched pathways were selected for further analysis.

### Immune infiltration analysis

To assess the immune microenvironment, the immune cell composition of the TME was evaluated using the GSVA algorithm. Differences of immune cell infiltration between these groups were analyzed, and statistical significance was determined using the Wilcoxon rank-sum test. Correlation analysis was also performed to investigate the relationship between the expression levels of the 20 candidate genes in the risk signature and the abundance of different immune cell types.

### Single-cell RNA sequencing analysis

In order to investigate cell composition and role in LUAD, we analyzed available scRNA-seq data (GSE210347). The data were processed using the Seurat package (version 4.0) to identify clusters of cells based on canonical marker expression. A total of 1,210 cells were clustered into eight major cell types: T cells, endothelial cells, fibroblasts, alveolar type II cells, neutrophils, macrophages, B cells, and epithelial cells.

### Trajectory and pathway enrichment analysis of macrophages

To investigate the differentiation trajectory of macrophages, the Monocle2 algorithm was used to reconstruct the evolutionary trajectory of macrophage subtypes. DEG between MS4A7^+^ macrophages and the other two macrophage subtypes (CCL18^+^ and FOLR3^+^) were identified using the DESeq2 package in R. Pathway enrichment analysis was performed using KEGG to identify biological processes associated with MS4A7^+^ macrophages.

## Metabolic profiling of macrophages

Metabolic pathways activated in MS4A7^+^ macrophages were identified using the scMetabolism algorithm, which enables the assessment of metabolic activity based on single-cell RNA-seq data. Comparative analysis of metabolic pathways was performed across the three macrophage subtypes (MS4A7^+^, CCL18^+^, and FOLR3^+^), focusing on pathways related to lipid metabolism, steroid biosynthesis, and ferroptosis regulation.

### Intercellular communication analysis

The CellChat algorithm was used to quantify intercellular communication networks within the TME. Receptor-ligand interactions were analyzed to identify communication axes between MS4A7^+^ macrophages and other immune cells, such as T cells and neutrophils. Interaction centrality was calculated to assess the relative importance of each immune cell subset in coordinating the immune response within the TME.

### Statistical analysis

Statistical analyses were conducted using R (version 4.0.3). All tests with a p-value <0.05 were considered statistically significant. Kaplan-Meier survival curves were compared using the log-rank test. To analyze immune infiltration, differences between high-risk and low-risk groups were assessed using the Wilcoxon rank-sum test, and Spearman’s rank correlation was used to determine correlations between gene expression levels and immune cell infiltration.

## Results

### Construction of a metabolism-related gene risk signature model in LUAD

**FIGURE 1:**
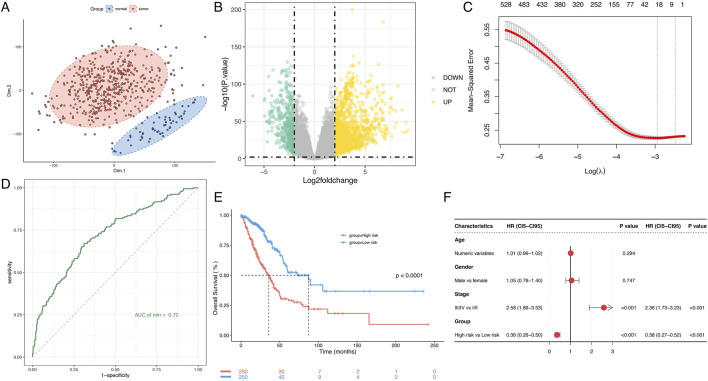
Prognostic value of the metabolism-related gene risk signature in LUAD. **(A)** PCA plot of LUAD samples based on the expression of metabolism-related genes. **(B)** Volcano plot of differential expression of metabolism-related genes between normal and tumor samples. **(C)** Lasso model identifies 20 genes as the metabolism-related gene signature. **(D)** ROC curve of the gene risk signature. **(E)** Kaplan-Meier survival analysis of OS in LUAD. **(F)** Univariate and multivariate Cox regression analyses to assess the prognostic significance. PCA, Principal component analysis; LUAD, Lung adenocarcinoma; ROC, Receiver Operating Characteristic; AUC, Area under the curve; OS, Overall survival.

RNA-seq data and clinical information from 500 LUAD patients were retrieved from TCGA. Following clustering analysis of normal and tumor tissues, metabolism associated DEG were identified by aligning them with the GeneCards metabolic database (Figures 1A, B). To explore the role and predictive potential of metabolism-related genes in LUAD, these metabolism associated DEG were included to LASSO-Cox regression analysis. By optimizing the penalty parameter (λ), 20 candidate genes were selected for constructing the prognostic model (Figure 1C; Supplementary Figure S1). The predictive performance of the model was evaluated by comparing the AUC of the ROC curves for the established model and each individual gene. The AUC for the integrated risk signature reached 0.72, significantly outperforming predictions based on single genes (Figure 1D, Supplementary Figure S2, S3). The median risk score was used to assign patients into high-risk and low-risk subgroups. Most of the selected genes showed significantly different expression levels between the high-risk and low-risk groups (Supplementary Figure S4). Kaplan-Meier survival analysis revealed a significant better overall survival in low risk group (P < 0.0001, Figure 1E). The multivariate Cox regression analysis confirmed that the risk score is an independent predictor of LUAD prognosis (Figure 1F), highlighting the model’s robust prognostic accuracy in stratifying LUAD patients.

### Comparative enrichment analysis and immune microenvironment characterization between these groups

**FIGURE 2:**
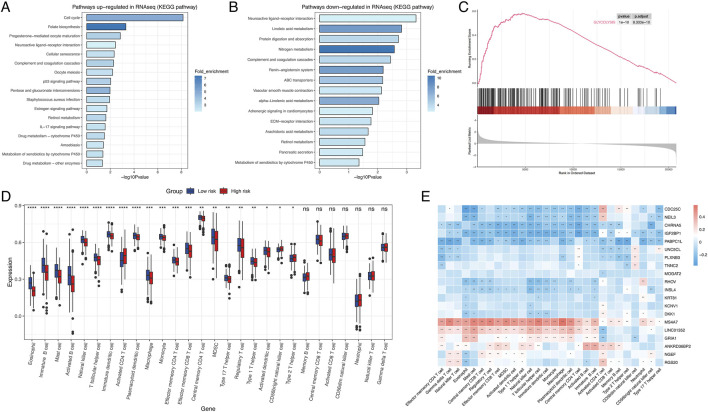
Transcriptomic pathway and immune-profiling analysis. **(A)** KEGG pathway upregulated in high risk group. **(B)** KEGG pathway downregulated in high risk group. **(C)** Glycolysis was upregulated in high risk group. **(D)** Boxplot of immune cell profiles **(D)** Dot plot highlighting differentially expressed genes (e.g., CDC25C, NEIL3, DKK1) associated with risk groups. Error bars represent standard deviation. **(E)** Heatmap displaying the correlation between genes and immune cell. *:P < 0.05; **P < 0.01; ***P < 0.001; ns, not significant.

To assess functional differences between these groups, KEGG pathway enrichment analysis revealed that the high-risk group showed significant upregulation of pathways related to cell cycle regulation, folate biosynthesis, p53 signaling, and drug metabolism (Figure 2A), which may contribute to their poorer prognosis. In contrast, neuroactive ligand-receptor interactions, linoleic acid metabolism, protein digestion/absorption, and nitrogen metabolism were downregulated in this cohort (Figure 2B). GSEA analysis further revealed pronounced activation of metabolic pathways in high-risk patients, including glycolysis, mTORC1 signaling, reactive oxygen species (ROS) production, cholesterol homeostasis, and hypoxia responses (Figure 2C; Supplementary Figure S5). Collectively, these findings suggest that dysregulated metabolic pathway activity significantly impacts LUAD prognosis.

To characterize TME heterogeneity, GSVA analysis was performed. The low-risk group exhibited significantly higher infiltration levels of eosinophils, immature B cells, mast cells, activated B cells, natural killer (NK) cells, macrophages, monocytes, effector memory CD4 T cells, effector memory CD8 T cells, and regulatory T cells. In contrast, activated CD4 T cells and type II T helper cells were enriched in the high-risk group. No statistically significant differences were observed between the groups for memory B cells, central memory CD8 T cells, activated CD8 T cells, CD56dim NK cells, neutrophils, natural killer T cells, and gamma delta T cells (Figure 2D). Next, we conducted correlation analyses between the expression levels of the 20 genes integrated into the signature and immune cells infiltration. The result demonstrated that both MS4A7 and LINC01352 showed strong correlations with most immune cell subtypes. Notably, MS4A7 displayed significant associations with all infiltrating immune cell populations except CD56dim NK cells (Figure 2E).

### MS4A7 as a key metabolism-associated gene in LUAD

**FIGURE 3:**
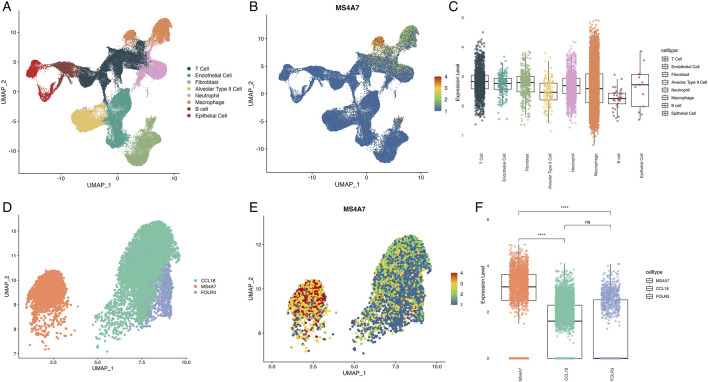
Single-cell RNA sequence analysis of cell distribution and MS4A7 expression. **(A)** UMAP plot of cell types in LUAD. **(B)** Expression of MS4A7. **(C)** Boxplot illustrating differential expression levels of MS4A7. **(D)** UMAP plot of macrophage subtypes. **(E)** Distribution of MS4A7 expression across various macrophage subtypes. **(F)** Boxplot illustrating differential expression levels of MS4A7. UMAP: Approximation and Projection.

To identify critical metabolism-related genes, we performed scRNA-seq on LUAD samples. A total of 1,210 cells were clustered into eight major cell types based on canonical marker expression: T cells, endothelial cells, fibroblasts, alveolar type II cells, neutrophils, macrophages, B cells, and epithelial cells (Figure 3A; Supplementary Figure S6). Among the 20 candidate genes, MS4A7 was the only gene with detectable expression, predominantly enriched in macrophage populations (Figure 3B; Supplementary Figure S7). Although no significant differences in MS4A7 expression were observed across heterogeneous cell subtypes (Figure 3C), this could be due to potential confounding effects from cellular heterogeneity. Therefore, we focused further analysis on macrophages. Macrophages were classified into three distinct subtypes based on their marker genes: CCL18^+^ macrophages (high CCL18), MS4A7^+^ macrophages (high MS4A7), and FOLR3^+^ macrophages (high FOLR3) (Figure 3D; Supplementary Figure S8). Expression profiling demonstrated ubiquitous MS4A7 distribution across all macrophage subsets, with the highest expression localized to the MS4A7^+^ subset (Figure 3E). These findings were validated through quantitative analysis, which showed a significant increase in MS4A7 transcript levels in the MS4A7^+^ macrophage subset when compared to the other two groups. (Figure 3F).

### MS4A7+ macrophages play a vital role in the TME of LUAD

**FIGURE 4:**
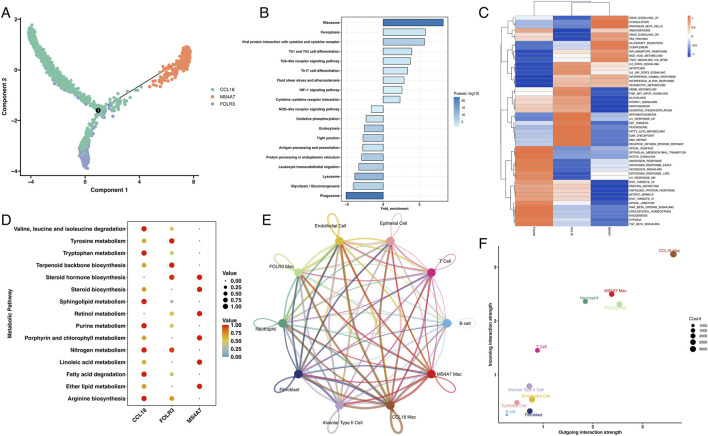
The role of MS4A7^+^ macrophage in LUAD. **(A)** The pseudotime analysis of macrophages. **(B)** KEGG analysis of enriched pathway in MS4A7^+^ macrophage. **(C)** GSVA of hallmarker genesets. **(D)** MS4A7^+^ macrophage displayed unique metabolism characteristic. **(E)** Cellchat analysis of different cells. **(F)** MS4A7^+^ macrophage displayed important role in tumor environment.

Given the unique expression pattern of MS4A7 in macrophages, we used the Monocle2 algorithm to reconstruct the evolutionary trajectory of macrophages. Trajectory analysis suggested that MS4A7^+^ macrophages likely represent terminal differentiation states originating from either CCL18^+^ or FOLR3^+^ macrophage precursors (Figure 4A). To further characterize the functional specialization of MS4A7^+^ macrophages, we conducted comparative functional enrichment analyses of DEG between MS4A7^+^ macrophages and the other two macrophage subpopulations. KEGG pathway analysis revealed significant enrichment of MS4A7^+^ macrophages for pathways related to ribosome biogenesis, ferroptosis regulation, viral protein-cytokine receptor interactions, and Th1/Th2 cell differentiation. Conversely, these cells displayed downregulation of pathways in phagosomal maturation, glycolysis/gluconeogenesis, lysosomal function, and leukocyte transendothelial migration (Figure 4B). GSVA using Hallmark gene sets further identified elevated activity in KRAS signaling, pancreatic β-cell function, angiogenic processes, p53-mediated apoptosis, and NF-κB-dependent TNF-α signaling pathways (Figure 4C).

Metabolic profiling using the scMetabolism algorithm revealed distinct metabolic reprogramming in MS4A7^+^ macrophages, with enhanced steroid hormone biosynthesis, retinol processing, and lipid metabolism pathways, including linoleic acid and ether lipid metabolism. Comparative analysis showed that CCL18^+^ macrophages preferentially activated branched-chain amino acid degradation and sphingolipid metabolism, while FOLR3^+^ macrophages upregulated nitrogen compound metabolism and terpenoid biosynthesis (Figure 4D). Additionally, CellChat analysis quantified intercellular communication networks, revealing that MS4A7^+^ macrophages exhibit broad interactivity within the TME, particularly with T cells and neutrophils (Figure 4E; Supplementary Figure S9). Receptor-ligand interaction analysis demonstrated that MS4A7^+^ macrophages engage in intercellular communication through distinct chemokine signaling axes. Specifically, these macrophages mediate crosstalk with T cells and neutrophils via the CCL3-CCR5 and CCL4-CCR5 ligand-receptor pairs, as evidenced by significant enrichment in these interaction modules. Furthermore, the CXCL12-CXCR4 signaling axis plays a key role in orchestrating MS4A7^+^ macrophage-T cell interactions (Supplementary Figure S10). Notably, all three macrophage subsets exhibited high interaction centrality, indicating their pivotal role in coordinating TME dynamics (Figure 4F).

## Discussion

Despite advancements in treatment, the prognosis for LUAD patients remains poor, especially those diagnosed at later stages (Schneider et al., 2020). As a result, identifying novel biomarkers to predict patient outcomes and guide treatment decisions is crucial. Among the emerging candidates, MRG have gained attention for their potential role in LUAD prognosis, reflecting the increasing recognition of metabolic reprogramming in cancer (Vander Heiden et al., 2009; Thesseling et al., 2020; Stine et al., 2022).

Cancer cells often exhibit altered metabolism to sustain rapid proliferation and survival within a challenging microenvironment (Ma et al., 2017; Shirakawa et al., 2017; Lim et al., 2020; Gheghiani et al., 2021). These metabolic changes result in the accumulation of metabolites in the TME, which can promote immune suppression. Consequently, MRGs are being explored not only as potential biomarkers but also as therapeutic targets. Recent studies have displayed that the reprogramming of metabolism in tumors plays a vital role in shaping immune composition of TME, influencing both tumor progression and immune responses.

Our research focuses on the prognostic importance of a metabolism-related gene risk signature in LUAD, constructed using data from 500 patients. This signature, developed through the LASSO-Cox regression model, consists of 20 candidate genes and has shown promise in predicting patient outcomes. Survival analysis indicated that high-risk patients experienced worse overall survival, reinforcing the utility of this model in clinical decision-making. Moreover, the risk score was confirmed as an independent prognostic factor, strengthening the robustness of the signature. The low-risk group exhibited a more favorable immune infiltration profile, with higher levels of immune cells: eosinophils, mast cells, activated T cells, and NK cells. On the other hand, the high-risk group showed elevated levels of immune suppressive cells, such as activated CD4 T cells and type II T helper cells, which are associated with immune evasion. These findings underscore the potential of metabolic reprogramming in influencing immune responses within the TME and its contribution to disease progression.

The role of MS4A7, a key gene within the metabolism-related signature, was further explored through single-cell RNA sequencing. MS4A7 was predominantly expressed in macrophages within the TME, a finding that highlights the importance of macrophages in shaping the immune microenvironment. Analysis identified three distinct macrophage subtypes—CCL18^+^, MS4A7^+^, and FOLR3^+^—with MS4A7^+^ macrophages exhibiting the highest expression of MS4A7. These cells are likely central to immune regulation within LUAD and may play a crucial role in modulating immune responses. In-depth analysis revealed that MS4A7^+^ macrophages are involved in key processes such as ribosome biogenesis, ferroptosis regulation, and T cell differentiation, suggesting their significant role in the immune microenvironment. Moreover, metabolic profiling of MS4A7^+^ macrophages indicated alterations in steroid hormone biosynthesis and lipid metabolism, which could contribute to the unique metabolic signature of the TME in LUAD. Intercellular communication analysis further emphasized the importance of MS4A7^+^ macrophages in mediating immune cell interactions within the TME, particularly through chemokine signaling axes. These interactions suggest that MS4A7^+^ macrophages play a pivotal role in immune modulation, influencing both tumor progression and therapeutic response. The clinical implications of these findings are twofold. Firstly, the metabolism-related gene risk signature, incorporating MS4A7, could act as an useful instrument for predicting patient prognosis. Secondly, the identification of MS4A7^+^ macrophages as a key component of the immune landscape opens up new therapeutic opportunities, particularly in the context of immunotherapy. Targeting MS4A7 or modulating macrophage function may enhance the anti-tumor immune response and improve patient outcomes.

However, there are limitations to this study. The reliance on retrospective data from TCGA and single-cell RNA-seq datasets restricts the ability to validate these findings in clinical settings. Further investigations are required to clarify the specific molecular mechanisms of MS4A7 in LUAD and to validate this risk signature in independent cohorts.

## Conclusion

In conclusion, our study highlights the critical role of metabolic reprogramming in LUAD and identifies MS4A7 as a potential biomarker and therapeutic target. The metabolism-related gene risk signature developed here provides a promising strategy for predicting patient outcomes. Future research focusing on the molecular mechanisms underlying MS4A7 expression will be essential for developing targeted therapies to improve outcomes for LUAD patients.

## Data Availability

The datasets presented in this study can be found in online repositories. The names of the repository/repositories and accession number(s) can be found in the article/Supplementary Material.
